# Waardenburg Syndrome: A Report of a Rare Case

**DOI:** 10.7759/cureus.65704

**Published:** 2024-07-29

**Authors:** Md Ilyaz, Renuka S Jadhav, Vineeta Pande, Shailaja Mane

**Affiliations:** 1 Pediatrics, Dr. D. Y. Patil Medical College, Hospital and Research Center, Dr. D. Y. Patil Vidyapeeth, Pune (Deemed to be University), Pune, IND

**Keywords:** dystopia canthorum, hearing loss, depigmentation of hairs, brilliant blue iris, waardenburg syndrome

## Abstract

Waardenburg syndrome (WS) is a rare autosomal genetic disorder characterized by oculocutaneous pigmentation defects, congenital deafness, dystopia canthorum, and a broad nasal root. It exhibits both genetic and clinical heterogeneity. This report presents a case of a 17-month-old male child who was brought to the Outpatient Department with complaints of hearing impairment and speech delay. Clinical examination revealed classic signs of WS, including iris pigmentary abnormality (brilliant blue iris), hair hypopigmentation (white forelock), dystopia canthorum, a broad nasal root, and a hypopigmented patch on the left arm. Based on the clinical features, the case was classified as WS Type I. This case underscores the importance of early recognition and diagnosis of WS for timely management and genetic counseling, particularly in pediatric patients with hearing impairment and distinctive pigmentation anomalies.

## Introduction

Waardenburg syndrome (WS) is a rare genetic disorder with an autosomal dominant inheritance pattern and is characterized by a group of distinctive features, including hair, skin, and eye pigmentation abnormalities, hearing loss, and facial anomalies [1]. WS was first described by Petrus Johannes Waardenburg, a Dutch ophthalmologist, in 1951. The prevalence of WS has been reported to be 1 in 40,000 births and is attributed to 2-5% of congenital hearing loss instances [2-4]. As an autosomal disorder, WS has a propensity to affect both males and females at a relatively equal rate. While WS is typically inherited in an autosomal dominant manner, there are instances where an autosomal recessive pattern of inheritance has been reported [5]. Patchy regions of depigmentation in WS are caused by an uneven scattering of melanocytes during embryogenesis. This condition is attributed to the degeneration of pigmentary cells in the stria vascularis, skin, hair, and eyes. This condition is characterized by several distinct clinical features. Patients typically exhibit a wide nasal root and lateral displacement of the medial canthi, often accompanied by the dystopia of the lacrimal puncta. There are also pigmentary anomalies of the iris, which can manifest as different-colored eyes or patches of color within the same iris. Hypertrichosis, or excessive hair growth, is often observed in the medial region of the eyebrows. A white forelock, a patch of white hair at the front of the scalp, is another hallmark feature. Additionally, affected individuals often experience deaf-mutism, a combination of congenital deafness and muteness, which significantly impacts communication and quality of life [3,4].

WS can arise from an array of genetic mutations encompassing insertion, deletion, frameshift, missense, and nonsense mutations. Among the four clinical variations, Types I and II are the most prevalent forms. Type I, caused by mutations in the paired box 3 (PAX3) gene, manifests as dystopia canthorum, congenital sensorineural deafness, neural tube abnormalities, cleft palate, and localized depigmentation of the skin and hair on the lip. The pigmentary defects of the eyes are linked to these symptoms. Type II is associated with mutations in the microphthalmia-associated transcription factor (MITF) gene [6]. The absence of dystopia canthorum differentiates Type II from Type I. Similar to Type I, mutations in PAX3 are also attributed to Type III WS. It exhibits combined characteristics of both Type I and Type II. However, with musculoskeletal anomalies such as upper limb abnormalities and shortened limbs, Type III WS is a severe form of Type I WS. Type IV involves mutations in the endothelin receptor type B (EDNRB) and endothelin 3 (EDN3) genes, which are typically autosomal recessive [7]. Given the genetic nature of WS, there is no specific known cure; however, supportive care options include cochlear implants and surgery if WS is associated with Hirschsprung syndrome. Genetic counseling must also be provided [8].

## Case presentation

**Figure 1:**
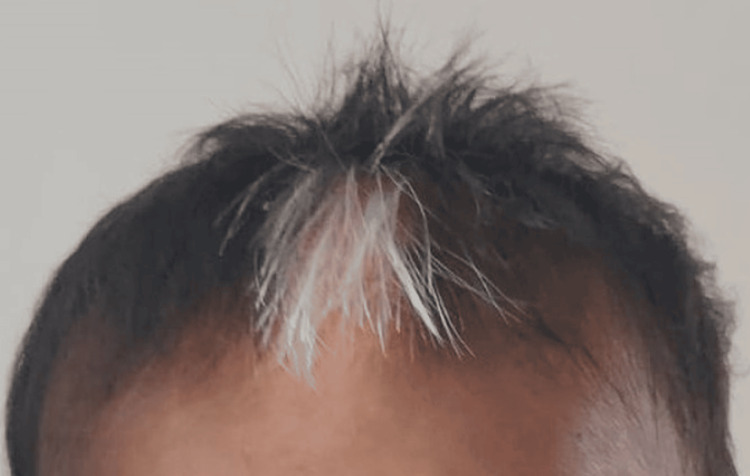
Clinical image of the patient A white forelock is present in the child

**Figure 2:**
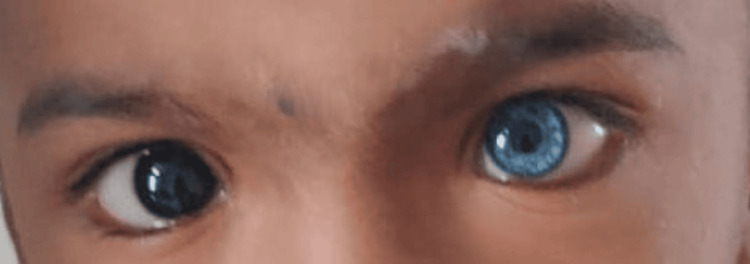
Brilliant blue iris (heterochromatic iris), wide nasal bridge

A 17-month-old male child was brought to OPD with complaints of hearing impairment and speech delay. He was found to have iris pigmentary abnormality (characteristic brilliant blue iris), hair hypopigmentation (white forelock), dystopia canthorum, and a broad nasal root (Figures 1-2). A hypopigmented patch of dimensions 3-4 cm on the left arm was also found. The child was born from a non-consanguineous marriage with no NICU stay. All developmental milestones were achieved up to age. The child was completely immunized up to nine months of age. Vitally stable, and neurological examination revealed no abnormal findings. Brainstem evoked response audiometry (BERA) investigation revealed bilateral profound sensorineural hearing loss. Based on clinical features, we classified this case as WS Type I. Following this diagnosis, genetic counseling was provided to the parents to explain the autosomal dominant inheritance pattern of the syndrome and to discuss the likelihood of their next child also being affected. A comprehensive treatment plan was developed, which included the recommendation for cochlear implantation to address the significant hearing impairment associated with the syndrome. The parents were thoroughly counseled on the benefits of cochlear implantation, including its potential to improve hearing and quality of life for their child.

## Discussion

WS is an autosomal dominant genetic disorder, and several hypotheses have been proposed to elucidate the diverse types, clinical manifestations, and underlying pathophysiology. According to a particular study, the syndrome is attributed to the anomalous formation of neural crest cells, resulting in defective neural crest. This theory proposes a connection between WS and Hirschsprung syndrome. Notably, the features of WS are also observed in the first arch syndrome [9,10]. Clinically, distinct postnatal morphological characteristics can be used to identify WS. The large nasal root, the variation in iris color, and the white forelock are characteristic traits of WS. Parents report that their child typically doesn't react to noises. Not all cases of WS display every clinical characteristic. The short philtrum, short retropositioned maxilla, large nasal root, and dystopia canthorum are the characteristics of Type I. Sensorineural deafness, differently colored irises, and normally positioned canthi are characteristics of patients with Type II WS [4,5]. The features of Type III WS, also known as Klein-Waardenburg syndrome, are similar to those of Type I WS, but there are more musculoskeletal abnormalities. These include hypoplasia of muscles with syndactyly, aplasia of the first and second ribs, aplasia of the arms, and small carpal bones that have not fully differentiated. In addition to having significant bone deformities, mental impairment, and microcephaly, some Type III patients have all key symptoms.

The characteristics of Type II and Type IV Williams syndromes are identical, except that Type IV WS (also known as Shah-Waardenburg syndrome) is associated with congenital megacolon (Hirschsprung disease) [7]. Skin, hair, and eyes are all impacted by WS pigmentary anomalies. Achromic patches and hyperpigmented macules over normal skin are symptoms of abnormal skin. Bilateral isohypochromia and heterochromia are examples of eye abnormalities. WS is clinically diagnosed using a set of major and minor criteria that aid in identifying the condition. The major criteria include sensorineural hearing loss, which affects the inner ear and the neural pathways to the brain; heterochromia (differences in iris color); a distinctive white forelock of hair; lateral displacement of the inner canthi of the eyes (giving a wider appearance); and having a first-degree relative with WS, indicating a genetic link. Minor criteria provide additional diagnostic support and include a broad nasal bridge, underdevelopment of the nasal alae, white patches or macules on the skin, synophrys (joined eyebrows), and premature greying of scalp hair. Two major criteria, or one major and two minor criteria, are required for the clinical diagnosis of Type I WS. To address hearing loss in Type I WS, cochlear implant surgery is utilized, and family members who may also have hearing loss are screened. Moreover, it has been recommended that pregnant women take folic acid supplements to reduce the chance of delivering a child with brain and spinal cord abnormalities.

## Conclusions

This case underscores the critical role of early diagnosis, genetic counseling, and appropriate intervention in managing genetic disorders and improving patient outcomes. Effective management of Type I WS involves a multidisciplinary approach, incorporating insights from otologists, ophthalmologists, dermatologists, and geneticists. By working together, these specialists can tailor interventions to the patient's specific needs, addressing both the hearing impairment and other associated features of the syndrome. A coordinated strategy not only aids in accurate diagnosis but also ensures comprehensive care that can significantly enhance the quality of life for the patient. In this instance, the recommendation for cochlear implantation exemplifies how targeted treatment can address significant auditory challenges, while genetic counseling provides essential information for future family planning. Overall, this integrated approach is crucial for optimizing care and outcomes in complex genetic conditions.
